# The influence of women’s groups mobilisation on health emergency response: Evidence from the Ebola outbreak in Liberia

**DOI:** 10.1371/journal.pone.0325496

**Published:** 2025-06-18

**Authors:** Florence Wullo Anfaara, Erica S. Lawson, Kilian Nasung Atuoye, Isaac Luginaah

**Affiliations:** 1 Department of Gender, Sexuality, and Women’s Studies, Western University, London, Ontario, Canada; 2 Department of Global Development Studies, Queen’s University, Kingston, Ontario, Canada; 3 Department of Geography and Environment, Western University, London, Ontario, Canada; Sergio Arouca National School of Public Health: Escola Nacional de Saude Publica, BRAZIL

## Abstract

**Background:**

The 2014–2016 Ebola outbreak in West Africa showed that multiple response strategies are necessary to contain disease outbreaks in resource-constrained settings. A critical component of these response strategies was the involvement of community members and women’s groups in leading them. While women’s groups actively participated in Ebola containment strategies in various communities, there is a dearth of research on their role or how their presence in communities affected the deployment of Ebola response strategies. In contributing to bridging this knowledge gap, we ask: How did the presence of women’s groups influence perceptions of Ebola response strategies in Ebola-affected communities in Liberia?.

**Methods:**

We fitted multivariate multinomial logistic regression models to cross-sectional data (n = 1,340) collected in five counties in Liberia. We built a composite model from community response strategies that participants reported witnessing. These responses were then categorized into no response, single-response, and multiple-response strategies. Response strategies included Ebola education and sensitisation campaigns, community surveillance, lobbying for personal protective equipment (PPEs), and other measures (e.g., prayer). Single responses pertain to participants choosing only one response strategy, while multiple responses indicate the selection of two or more response strategies.

**Results:**

Overall, we found that knowledge about the presence of women’s groups was associated with an increased likelihood of participants reporting having witnessed a community response during the Ebola outbreak. Specifically, participants who reported having women’s groups in their communities had 89% and 98% higher odds of reporting a single Ebola response (RRR = 1.89, p ≤ 0.001) and multiple responses (RRR = 1.98, p ≤ 0.001), respectively. We also found some demographic, socioeconomic, and place-based variables to be associated with Ebola response strategies.

**Conclusion:**

We provide relevant policy recommendations necessary to center women’s groups and other community organisations in Liberia’s strategic health plan toward a pandemic-ready future. We believe that strengthening local, national, and international collaborations is critical to achieving this future and can help the country reach its SDG 3.3 goal of ending infectious diseases by 2030.

## Introduction

With over 28000 cases and 11000 deaths, the 2014–2016 Ebola epidemic in West Africa was declared by the World Health Organization (WHO) as a public health emergency of international concern and a threat to global security and international peace [1]. Liberia was the most affected, recording 37% and 42% of total cases and deaths, respectively [2]. Some reasons posited for the outbreak of Ebola in Liberia have been associated with the decimation of the healthcare system and underdevelopment resulting from the country’s 14-year civil war [3–5], mistrust of government and state institutions [6–8], and the slow response from the WHO and the international community [9–11]. Before the Ebola outbreak in Liberia, the country’s healthcare system was still recovering from the 14-year civil war and was, therefore, inadequate to handle the transmission and death rates. Fewer and ill-equipped health infrastructure and inadequate medical personnel meant that public health interventions to contain the Ebola epidemic rested on collaborative efforts of the community, government, and international organizations [4,12].

International response strategies by the WHO, the United States Centers for Disease Control and Prevention (CDC), and other international community members provided financial and technical support to the Liberian government. These included devising and implementing multiple response strategies, such as rigorously implementing the WHO-recommended non-pharmaceutical interventions (e.g., case isolation, quarantine, encouraging sanitary practices of washing hands and burial, and social distancing) [13]. Non-pharmaceutical interventions have proven effective in controlling disease spread, as showcased during Ebola and the recent COVID-19 pandemic [14].

In addition to the international response, local community members, including women’s groups, were particularly influential during the all-hands-on-deck Ebola response systems. Community leaders leveraged their influence, social networks, and community mobilization to implement community-level Ebola containment strategies that have been credited with helping to end Ebola in Liberia. In particular, women’s groups have led and engaged in Ebola containment practices by devising locally made equipment and using it to manage Ebola victims in families and communities [15]. Women’s groups served as contact tracers and case finders in their communities. They engaged in Ebola education and sensitisation programs through house-to-house campaigns and radio/town crier services to raise awareness of Ebola safe practices [15]. Indeed, Sharon Abramowitz and colleagues [16] have discussed the gendered labour distribution of Ebola prevention in communities, highlighting how women’s groups supported community mobilisation efforts by screening for Ebola symptoms and enforcing Ebola quarantine measures. Perry [1] also described how a female chief mobilised her community to build a 3-bedroom structure to care for Ebola victims. Further, Leymah Gbowee and Ruth Caesar, who have both played critical roles in forming women’s groups in Liberia, documented the various caregiving roles women’s groups played as first responders during the Ebola epidemic in Liberia [17,18].

Despite the significant contribution of women’s mobilisation efforts to ending Ebola, much has not been discussed on how community members perceived their presence and roles in helping manage the Ebola crisis in the country. Additionally, the current literature seems to credit community male leaders and external partners [19,20] without fully exploring how residents linked the presence of women’s groups to Ebola mitigation efforts in their respective communities. To contribute to bridging this knowledge gap, we pose the following research question: How did the presence of women’s groups influence perceptions of Ebola response strategies in Ebola-affected communities in Liberia? We hope that the findings from this study will contribute to Liberia’s pandemic preparedness efforts and the broader discussion of achieving SDG 3.3, which aims to end infectious diseases in Liberia and globally.

### Theoretical context: community mobilisation theory

Rooted in social movement theory, community mobilisation (CM) generally incorporates community engagement, solidarity, and trust-based social networks to implement a mutually beneficial collective action by a group, society, or population toward a common interest [21,22]. Within health promotion and public health discourses, community mobilising strategies involve people organising material and non-material resources to address life-threatening crises affecting their health and well-being [21]. In the context of disease outbreaks and pandemics, CM approaches involve community engagement and working collaboratively with local and international institutions, stakeholders, and partners to mobilise resources for disease containment [22]. CM focuses on bottom-up approaches that empower individuals and communities to take charge of their health through self-initiated and self-directed interventions [21,23]. Scholarship around CM encompasses social mobilisation and community-based approaches to health promotion, including those that point to the efficacy of CM in influencing the uptake of HIV testing [24,25], COVID-19 containment [26], improving antenatal and postnatal care uptake [27,28], gender equality and prevention of violence against women [29,30].

For CM strategies to effectively influence positive public health behaviour, mobilising groups must be perceived as trustworthy within their social and community networks. (Mis)Trust can promote or mar an intervention in emergency situations, as reflected in the Liberian context. Decades of governmental and institutional distrust resulting from corruption, civil war, and state abuse led to public distrust of the Liberian government [4] and its Ebola control efforts. Lack of trust, distrust, and suspicion of the state led people to believe that Ebola was a ploy by the Liberian government to solicit more financial aid from international organisations and subsequent resistance of the public to follow safe burial practices [6,7,31], significantly affecting the spread of Ebola in the early days of the Liberian outbreak. Implementing community engagement strategies at the midpoint of the Ebola outbreak through collaborations and partnerships with community leaders and community-based organisations, including women’s groups, changed the tide of Ebola containment in Liberia. With more community involvement came increased compliance with Ebola interventions. Citizens’ trust in existing relationships with community-based organisations made compliance easier [7]. In particular, women’s groups have built trust-based relationships with the communities in which they work. Their earlier involvement with the Liberia Mass Action for Peace Campaign and the Accra peace processes has given them social and cultural respectability locally and internationally, such that their presence and participation in an intervention may garner support and compliance. The few available studies that have documented women’s Ebola mobilisation strategies have suggested how Liberia women view Ebola work as an extension of their social reproductive and peacebuilding roles, whether in taking care of sick family members and leading Ebola education campaigns [10,15,16] or in mobilising community members to build local quarantine centers [1]. These community mobilization strategies would not have been possible without the social networks and trust-based relationships women’s groups have in the communities. As such, we expect that communities with women’s groups will have a positive perception of Ebola responses in their communities.

Furthermore, studies in public health research have demonstrated how differences in backgrounds can influence people’s perceptions of disease containment response strategies. These differences may be grouped into demographic, socioeconomic, and place-based factors. For instance, in the recent COVID-19 pandemic, studies across varying contexts established that adherence to health interventions was influenced by demographic characteristics such as age, gender, marital status, and religion [32–34]. Similarly, socioeconomic factors such as education, occupation, and household wealth [35,36], as well as location (rural/urban and county of residence), can affect how people perceive the presence of health interventions during a disease outbreak [37,38]. Consequently, we expect to see how these factors affect participants’ perceptions of Ebola response strategies in Liberia.

## Materials and methods

### Data

#### Sampling strategy.

This study used quantitative data from a larger study to assess the influence of women’s groups in containing Ebola in Liberia. We conducted the survey research from April to August 2022 during COVID-19, when travel restrictions were in place. We adopted the multi-stage sampling design that follows the Liberia Institute of Statistics and Geo-Information Services (LISGIS) 2008 Population and Housing Census sampling procedure [39]. First, we identified five of the fifteen counties that were most affected by Ebola (i.e., Montserrado, Bong, Bomi, Margibi, and Lofa,). Next, we selected twenty districts and thirty-nine communities and interviewed 1400 households. Sample size distribution across the five counties was based on probability propoortional to size and obtained from the list of enumeration areas and communities used in the 2008 Population and Housing Census [39]; as such, counties with larger populations (e.g., Montserrado) will have a larger sample distribution. In the final stage, we employed the following steps to randomise our household selection in each community. Most settlement/housing structures in the study communities are arranged linearly, with paths or roads separating them, making randomization easy. For each community, linear settlements are divided into Line 1 and Line 2. For Line 1, the first house was selected as the starting point, and then we counted and selected every third house; b) In Line 2, we started with the second house and then selected every fourth house. These steps were repeated until we arrived at our desired sample for each community. For each house, the household head, their spouse/partner, or an elderly person was interviewed. Participants were ineligible to participate if they were under 18 or did not provide verbal consent. Slovin’s sample size formula ([n = N/(1 + (N*e^2))] was used as a guide to determine the sample size for this study. A 95% confidence interval and 3% margin of error yielded a minimum required sample size of 1110. However, we increased the sample size to 1400 to increase statistical power during analysis. Data cleaning yielded an analytical sample of 1,340. Ethical approval was obtained from the Western University Non-Medical Research Ethics Board (NMREB).

#### Data collection procedure.

Qualtrics was used to design our survey instrument. During the COVID-19 pandemic, Qualtrics’ data collection tool was deemed the most appropriate method for conducting survey research because of its versatility, data quality, and cost-effectiveness [40]. We adapted standardized questions on Ebola knowledge, attitudes, and practices from the Liberian Ministry of Health’s nationally representative study on Ebola [41]. We also included survey questions informed by the research objectives on women’s grassroots contributions to managing Ebola in communities. To help us collect quality data, we recruited and trained twelve experienced enumerators with skills, expertise, and knowledge about the study sites. Enumerators had college or university degrees, lived in the study sites, worked in pairs, and attended a three-day Zoom training workshop on research ethics, particularly on participants’ privacy and confidentiality. Additionally, enumerators were trained on using Qualtrics and had the opportunity to pretest the survey tool before heading to the field. While the research team, including the lead author on this paper, supervised data collection remotely, the twelve enumerators conducted the survey interviews in person using the tablets preloaded with the Qualtrics questionnaire shipped to them from Canada. Each enumerator was given a daily survey target (ranging from 5–7, depending on the County size). Internet data was provided to each enumerator to ensure they uploaded completed surveys daily. As a survey tool, Qualtrics has built-in tools that track and collect geographic data of study sites, which is an important component of ensuring the quality of the data. Another step in ensuring data quality was having a two-step supervision of all data collected. A field supervisor checked daily on enumerators in person and on the phone while the research team monitored the daily data uploaded to the server for errors or omissions. We cleaned the data and created codes in Excel before exporting the data into STATA for subsequent statistical analyses.

### Measures

Our outcome variable, ‘Community response strategies to Ebola,’ was derived from the question, ‘How did your community respond to the 2014 Ebola outbreak? Participants could choose one or multiple response strategies as needed, including a) my community did nothing, b) Ebola education/awareness, c) community surveillance, d) lobby for Ebola prevention supplies, and e) Other, in which case the participant specified. We constructed the ‘community response’ variable and created three categories (i.e., no response, single response, and multiple response see Table 1 for percentage distribution). *Single responses* category indicates participants choosing only one of the above options (e.g., education, community surveillance, lobbying, or other), while those that selected two or more options (e.g., education and community surveillance or education, surveillance, lobbying) were categorized as *multiple responses*. “My community did nothing” was categorized as *no response*. We coded responses as follows: no response = 0, single response = 1, and multiple responses = *2*.

**Table 1:** Sample characteristics.

	Percentage (n = 1340)
**Ebola response strategies**	
No response	16
Single response	44
Multiple responses	40
**Women groups**	
No	52
Yes	48
**Gender**	
Female	69
Male	31
**Age**	
19-28	20
29-38	35
39-48	26
49+	19
**Marital Status**	
Married	30
Never married	55
Formerly married	15
**Religion**	
Christian	81
Muslim	17
Traditional	2
**Wealth Quintile**	
Poorest	20
Poor	20
Middle	20
Richer	20
Richest	20
**Education**	
No formal edu.	23
Primary	29
High school	30
Tertiary	18
**Occupation**	
Others	8
Self-employed	8
Petty trading	53
Public Service	18
Farming	13
**Presence of health facility**	
No	18
Yes	82
**Rural/Urban**	
Rural	27
Urban	73
**County**	
Bong	15
Bomi	7
Lofa	14
Margibi	15
Montserrado	49

n, analytical sample.

The key independent variable, ‘women’s groups,’ came from the question, ‘Do you have women’s groups in this community?’ Responses were coded as follows: no, 0, and yes, 1. We added other theoretically relevant control variables specific to the study context, including demographic, socioeconomic, and place-based variables. The demographic variables included in this study were gender, age, marital status, and religion. Education, occupation, and household wealth index constituted the socioeconomic variables. Following the criteria used by the Demographic and Health Survey (DHS) conducted in Liberia and other African contexts [42–44], we collected variables on the economic conditions of participants, such as household assets like radio and television, electricity, access to running water, ownership of livestock, and many others. Using principal component analysis, we constructed a composite variable from these responses and recategorized it into five wealth categories (i.e., poorest, poor, middle, richer, richest). Reporting household income this way provides a fairly accurate representation of living conditions. Finally, location and place-based variables included rural/urban status, county of residence, and the presence of a health facility. All variables with a value of 1 in Tables 2 and 3 serve as the reference category against which we tested our outcome variable in the analyses. Table 1 contains the sample distribution and characteristics of the control variables.

**Table 2:** Bivariate multinomial logit models estimating women’s groups’ influence on Ebola epidemic response in Liberia.

	Single response		Multiple response	
Variables	RRR (Robust std. err)	95% CI	RRR (Robust std. err)	95% CI
**Women group**				
No	1		1	
Yes	1.63 (0.25)***	1.21-2.22	1.33 (0.21)	0.98-1.81
**Gender**				
Female	1		1	
Male	0.80 (0.13)	0.58-1.10	0.76 (0.12)	0.55-1.05
**Age**				
19-28	1		1	
29-38	0.52 (0.12)**	0.32-0.84	0.53 (0.12)**	0.32-0.85
39-48	0.54 (0.13)*	0.33-0.89	0.41 (0.10)***	0.25-0.69
49+	0.33 (0.08)***	0.19-0.55	0.43 (0.11)***	0.26-0.72
**Marital status**				
Married	1		1	
Never married	1.53 (0.28)*	1.07-2.19	0.87 (0.15)	0.61-1.24
Formerly married	0.58 (0.13)*	0.37-0.90	0.36 (0.08)***	0.23-0.57
**Religion**				
Christianity	1		1	
Muslim	0.71 (0.15)	0.47-1.09	1.27 (0.26)	0.85-1.90
Traditional	0.16 (0.07)***	0.06-0.41	0.14 (0.07)***	0.05-0.39
**Wealth Quintile**				
Poorest	1		1	
Poorer	0.80 (0.23)	0.46-1.41	1.27 (0.38)	0.70-2.29
Middle	0.24 (0.07)***	0.14-0.42	0.95 (0.25)	0.56-1.61
Richer	0.23 (0.06)***	0.14-0.39	0.57 (0.15)*	0.34-0.96
Richest	0.31 (0.08)***	0.18-0.51	0.55 (0.15)*	0.32-0.95
**Education**				
No formal edu	1		1	
Primary	4.25 (0.99)***	2.69-6.72	3.83 (0.90)***	2.41-6.09
High school	2.19 (0.45)***	1.45-3.31	2.52 (0.53)***	1.67-3.81
Tertiary	1.01 (0.21)	0.66-1.54	0.91 (0.20)	0.59-1.41
**Occupation**				
Other	1		1	
Self-employed	0.37 (.16)*	0.15-0.87	0.28 (0.12)**	0.12-0.65
Petty trading	0.47 (.17)*	0.23-0.97	0.30 (0.10)***	0.14-0.60
Public service	1.02 (.42)	0.46-2.29	0.52 (0.21)	0.23-1.17
Farming	0.40 (.16)*	0.18-0.90	0.32 (0.13)**	0.15-0.71
**Rural-Urban**				
Rural	1		1	
Urban	0.75 (0.14)	0.53-1.08	0.69 (0.13)*	0.49-0.98
**Presence of health facility**				
No	1		1	
Yes	1.83 (0.34)***	1.28-2.64	1.62 (0.29)**	1.12-2.32
**County**				
Bong	1		1	
Bomi	4.36 (2.88)*	1.20-15.92	0.01 (0.04)***	0.00-0.11
Lofa	0.29 (0.13)**	0.11-0.71	0.11(0.04)***	0.05-0.26
Margibi	5.42 (3.90)*	1.32-22.24	1.69 (1.18)	0.42-6.69
Montserrado	0.33 (0.14)**	0.14-0.77	0.03 (0.01)***	0.02-0.07

**Note:** Base, no Ebola response; RRR, relative risk ratio; CI, confidence interval, *** p < 0.001, ** p < 0.01, * p < 0.05.

**Table 3:** Multivariate multinomial logit regression estimating women’s groups’ influence on the Ebola epidemic response in Liberia.

	Single response		Multiple responses	
Variables	RRR (Robust std. err)	95% CI	RRR (Robust std. err)	95% CI
**Women group**				
No	1		1	
Yes	1.89 (0.35)***	1.31-2.73	1.98 (0.39)***	1.34-2.92
**Gender**				
Female	1		1	
Male	0.91 (0.18)	0.61-1.35	0.59 (0.12)**	0.38-0.89
**Age**				
19-28	1		1	
29-38	0.71 (0.19)	0.41-1.22	0.44 (0.12)**	0.25-0.77
39-48	0.68 (0.20)	0.37- 1.22	0.46 (0.14)**	0.25-0.85
49+	0.50 (0.16)*	0.25-0.97	0.66 (0.23)	0.33-1.31
**Marital status**				
Married	1		1	
Never married	1.15 (0.26)	0.72-1.81	1.03 (0.25)	0.64-1.68
Formerly married	0.78 (0.20)	0.46-1.32	0.44 (0.12)**	0.25-0.78
**Religion**				
Christianity	1		1	
Muslim	0.61 (0.17)	0.35-1.07	1.12 (0.32)	0.64-1.97
Traditional	0.16 (0.09)**	0.05-0.50	0.20 (0.12)**	0.06-0.66
**Wealth Quintile**				
Poorest	1		1	
Poorer	0.60 (0.19)	0.31-1.15	0.75 (0.27)	0.37-1.52
Middle	0.21 (0.07)***	0.11-0.39	0.90 (0.31)	0.46-1.77
Richer	0.18 (0.06)***	0.09-0.35	0.60 (0.21)	0.29-1.21
Richest	0.29 (0.10)***	0.14-0.57	0.64 (0.24)	0.30-1.37
**Education**				
No formal edu	1		1	
Primary	3.13 (0.84)***	1.84-5.31	3.03 (0.86)***	1.73-5.30
High school	1.90 (0.50)*	1.13-3.19	2.15 (0.60)**	1.24-3.71
Tertiary	1.08 (0.31)	0.61-1.92	1.06 (0.34)	0.56-1.99
**Occupation**				
Other	1		1	
Self-employed	0.55 (0.26)	0.21-1.43	0.49 (0.25)	0.18-1.33
Petty trading	0.41 (0.16)*	0.18-0.91	0.42 (0.17)	0.19-0.95
Public service	1.28 (0.58)	0.52-3.14	1.10 (0.52)	0.43-2.80
Farming	0.57 (0..27	0.23-1.45	0.39 (0.18)*	0.15-1.01
**Rural-Urban**				
Rural	1		1	
Urban	5.43 (1.78)***	2.85-10.34	2.84 (0.91)***	1.50-5.35
**Presence of health facility**				
No	1		1	
Yes	1.56 (0.33)*	1.02-2.38	1.75 (0.41)*	1.11-2.78
**County**				
Bong	1		1	
Bomi	8.42 (6.03)**	2.07-34.30	0.01 (0.01)***	0.00-0.11
Lofa	0.94 (0.48)	0.34-2.58	0.22 (0.10)**	0.08-0.58
Margibi	10.35 (7.82)**	2.35-45.53	2.15 (1.58)	0.50-9.14
Montserrado	0.30 (0.14)*	0.12-0.77	0.02 (0.01)***	0.01-0.05
**Log likelihood**	**−1044.3526**			
**LR chi2**	**702.54**			
**Pseudo R2**	**0.2517**			
**Number of observations**	**1,340**			

**Note:** Base, no Ebola response; OR, odds ratio; CI, confidence interval; *** p < 0.001, ** p < 0.01, * p < 0.05.

### Analyses

Our statistical analyses was done using STATA 18. First, we conducted a descriptive analysis of all variables to understand the distribution of these variables within the sample. Second, we ran a bivariate multinomial analysis to help us determine the independent relationship between our outcome variable (i.e., Ebola response strategies) and each covariate. After this, we ran a multivariate multinomial logistic regression. Multivariate analysis provides insight into the combined, simultaneous effect of all covariates on the outcome variable. This allows the changes and patterns of the multiple variables to be observed. We chose multinomial logistic regression (MLR) because the outcome variable has three categories (i.e., no response, single response, multiple responses). Before settling on MLR, we ran diagnostic tests for ordered logistic regression analysis to assess the suitability of our outcome variable to meet the proportional odds assumption [45]. The Brant test analysis we employed yielded a significant result (χ² = 194, p ≤ 0.001, df = 26), indicating that the proportional odds assumption was violated [45]. However, the test for independence of irrelevant alternatives (IIA) assumption necessary for running MLR was satisfied (Hausman test p > 0.05, not significant) [46]. As such, we employed MLR. Unlike ordered logistic regression, MLR provides alternatives to testing any data distribution’s correctness and model fitness regardless of those statistical assumptions [47]. We determined the model fitness for our mlogit analysis using *Fitstat*. To facilitate the interpretation of our results, we exponentiated the coefficient of the mlogit to obtain relative risk ratios (RRR) interpreted similarly to odds ratios. Therefore, results are presented in RRR and confidence intervals. As such, an RRR greater than 1 (RRR > 1) indicates a positive association and higher odds of predicting an Ebola response strategy. Conversely, RRR less than 1 (RRR < 1) indicates a negative association and a lower likelihood.

## Results

### Sample characteristics

Table 1 presents the sample characteristics of our data. Cumulatively, 84% of respondents reported witnessing an Ebola response strategy (i.e., single response (44%) and multiple response (40%)). While 48% reported having women’s groups in their communities, 52% reported otherwise. More than half of the respondents were female (69%), never married (55%), Christian (81%), engaged in petty trading (53%), resided in urban areas (73%), and had a health facility in their communities (82%). The majority live in Montserrado County (49%), are between the ages of 29 and 38 (35%), and have at least a primary education (29%) and a high school education (30%).

### Bivariate results

Results for the MLR analyses at the bivariate and multivariate levels are presented in Tables 2 and 3. Given that our outcome variable has three categories, with *‘no response’* serving as the base referent category, the results should be interpreted in relation to this base category (i.e., single response vs. no response and multiple response vs*.* no response). However, for clarity and to avoid repetition, the interpretations of the results below are limited to the two categories (i.e., single response and multiple response) for both bivariate and multivariate results.

Table 2 presents the unadjusted association between the outcome variable, the key independent variable, and the control variables. At the bivariate level, the outcome variable (i.e., Ebola response strategies) and the key independent variable (i.e., women’s groups) showed a statistically significant positive relationship for single responses (RRR = 1.63, p ≤ 0.001), but not for multiple responses.

In contrast, the relationship between the outcome and control variables was mixed, showing positive and negative associations for single and multiple responses. For instance, the education and health facility variables exhibited a statistically significant positive relationship for both single- and multiple-response strategies. Specifically, individuals with primary and high school education reported higher odds of experiencing both single (RRR = 4.25, p ≤ 0.001) and multiple response strategies (RRR = 3.83, p ≤ 0.001), respectively. Similarly, people who reported having a health facility in their communities were 1.83 and 1.62 times more likely to report higher odds of experiencing single and multiple response strategies, respectively. Conversely, age, religion, wealth, and occupation categories showed statistically negative relationships with the outcome variable.

### Multivariate results

Table 3 shows the results of our multivariate mlogit analyses. The results show the change in the association between the outcome variable and the focal independent variable (i.e., women’s groups) and the effect of other control variables. The first change is observed in our key independent variable, which shows that individuals living in communities with women’s group were 89% and 98% more likely to report higher odds of witnessing a single Ebola response strategy (RRR = 1.89, p ≤ 0.001) and multiple responses (RRR = 1.98, p ≤ 0.001), respectively. Participants in urban areas reported 5.43 times and 2.84 times higher odds of experiencing single and multiple Ebola response strategies, respectively. While gender was not significant in the bivariate results, the relationship changed in the multivariate results, with men reporting lower odds of experiencing multiple Ebola response strategies (RRR = 0.59 ≤ 0.01). The other control variables showed statistically significant negative associations, including wealth quintile, occupation, marital status, and age. However, the associations between the outcome variable and county of residence, education, religion, and presence of a health facility remained unchanged from the bivariate results.

## Discussion

This paper explored the perceived influence of women’s groups’ mobilisation initiatives in response to the Ebola outbreak in Liberia. Overall, we found that knowledge of the presence of women’s groups in communities affected people’s perception of reporting an Ebola response. Community response to Ebola was either single (i.e., one intervention only) and/or multiple (i.e., two or more interventions). Participants in communities with women’s groups reported higher odds of observing both single and multiple responses. This finding aligns with calls for integrating health equity and community engagement in public health interventions during epidemics and pandemics, especially in resource-constrained post-conflict contexts [48,49]. Two possible explanations can be provided for this observed relationship. The first is the widespread recognition of Liberian women as peacebuilders and gender equality advocates locally and internationally, resulting from their decades-old use of maternal and social activism [50,51], and the second is the trust-based relationships women’s groups have built over the years with communities [52,53]. We believe that because of the social and cultural visibility of women’s groups, their presence may have brought the spotlight to those communities, affecting the level of support communities received from national and international Ebola response teams. A recent study by Anfaara et al. [15] reported how women’s groups formed partnerships with national and international organisations (e.g., UN Women) to implement Ebola interventions in affected communities. Women’s groups reported receiving funds from these organisations to purchase and distribute Ebola supplies during the Ebola outbreak, including hand sanitisers and handwashing equipment. While others reported receiving technical training as contact tracers to help identify Ebola patients in their communities. These activities might have increased their visibility in communities, thereby influencing how community members perceived their role in Ebola strategies. Additionally, it is possible that women’s groups’ contribution to peace and development in Liberia gave them an added advantage as trustworthy community partners, contributing to adherence and compliance with the Ebola control measures. Research on community engagement, trust, and compliance with public health measures during disease outbreaks and pandemics has been widely documented [3,6,7,13].

Demographic characteristics, such as gender, age, marital status, and religion, affected how participants perceived Ebola response strategies in their communities. Men reporting lower odds for multiple responses corroborate with the literature about the gendered involvement and impacts of the Ebola outbreak in Liberia. Several studies have documented the burden of care work on women and how women’s caregiving duties made them susceptible to Ebola infection [10,16,54], as well as the social and economic impacts women faced at the peak of the epidemic [55]. We believe that the gendered implications of Ebola care work and social burdens on women may have led to targeted interventions for women as a group compared to men. For instance, targeted interventions aimed at increasing antenatal access and utilization for pregnant women during the Ebola outbreak have been reported [56,57].

Furthermore, people in the 29–48 age groups also reported lower odds of witnessing multiple responses. We explain this finding within the contextual dynamic of age-related roles and responsibilities of Liberians. Like elsewhere in Africa, Liberian adults within the 29–48 age categories are mostly the main providers and caretakers of their nuclear and extended families, who may have borne the brunt of providing for their families during Ebola [58]. While the working-age category ranges between 15 and 65 years in most developed countries, the situation in Liberia is quite different. The country is relatively young, with about 89% dependency ratio relative to the working population [59]. With a high dependency ratio, pressure to financially support dependent family members may have been placed on the 29–48 age cohorts who are within the active working population. We argue that taking care of bread-and-butter issues and Ebola-related medical costs might have been the primary goal of these adults during Ebola; evidence suggests that disruptions in economic activities heightened the suffering and poverty levels of the working few [60,61]. It is possible that people within the 29–48 age groups were preoccupied with providing for their families; as such, any intervention that was not financial may not have been perceived as an Ebola response for these people. For instance, Pellecchia et al. [62] have documented how the Liberian government and its international partners initiated an economic support package for poor households. This partially explains why people 49 and older may have indicated witnessing a single response.

Similarly, people who were formerly married reported lower odds for multiple responses, while those who identified with traditional religion reported lower odds for single and multiple responses. Health interventions, including outbreak response in SSA contexts and Liberia, tend to favour those in marriage unions. Legal unions, such as marriage or common law, are often considered the primary unit for deploying health interventions due to the likelihood of influencing positive behaviour change or compliance with a health intervention [32,63]. However, the unintended consequence is the potential for unmarried or formerly married individuals to be overlooked. During the Ebola outbreak in Liberia, for instance, while interventions focused on reducing Ebola spread through safer household dynamics and the impact of Ebola on increasing sexual and gender-based violence cases among married people [55], interventions targeting formerly married people were limited to social assistance, including food coupons and financial assistance, especially in rural areas [64,65]. This may have resulted in formerly married people reporting lower odds for multiple responses.

Regarding religious differences, it is documented that closing religious centres and banning some traditional practices was effective in controlling the spread of Ebola in affected communities [66]. Research shows that traditional beliefs and misconceptions about Ebola and unsafe burial practices greatly affected the spread of Ebola in Liberia [67]. Consequently, rigorous education and awareness campaigns on disease etiology and prevention, safe burial practices, and social distancing targeted religious groups, including those of traditional religion who predominantly congregate in specific geographic areas. Therefore, participants reported single or multiple responses depending on the religious activities and location, as highlighted by our findings.

Socioeconomic factors, including education, household wealth, and occupation, affected perceptions about Ebola responses. Except for education, people in all wealth categories, petty traders, and farmers reported lower odds for an Ebola response. As alluded to, the economic impact of Ebola exacerbated the living conditions of the poorest socioeconomic groups, most of whom are either employed in the informal sector as petty traders or in the agricultural sector as farmers [55]. The relationship between household wealth and occupation explains why the economic impact of Ebola in Liberia was pronounced. With most of Liberia’s economy relying on the informal sector, Ebola prevention strategies such as quarantine and social distancing affected markets where the majority of workers do business, thereby exacerbating the poverty situation in the country. We posit that interventions targeting this demographic may not have been sufficient, which may be reflected in their response. The education variable was significant for people with primary and high school education, who reported higher odds for both single and multiple Ebola responses. While this finding may seem counterintuitive and require further investigation, a possible explanation for this relationship may involve how public health interventions work in Africa. Most health interventions target people with lower educational status because of their perceived increased risk of non-compliance with safety measures [43,68]; consequently, Ebola response interventions may have specifically targeted these groups, resulting in how they perceive the response interventions.

Our location and place-based variables yielded some important findings that support the notion that geography tends to influence perceptions of health interventions. Communities with a health facility and people living in urban areas reported higher odds for single and multiple responses, respectively. In the context of the outbreak in Liberia, most health facilities were overcrowded as healthcare professionals prioritised attending to Ebola victims [69]. People who had access to health facilities or lived close to them would experience multiple Ebola responses, either through their role as healthcare recipients or targets of Ebola education and prevention strategies. Residents may have perceived the activities in and around healthcare facilities as response strategies. Likewise, due to the populous urban settlements in Liberia, urban dwellers may have received much attention regarding the Ebola response, owing to the urban bias in the delivery of healthcare services [70]. For instance, West Point, the biggest urban slum in Monrovia, was a target for multiple Ebola responses sometimes resulting in clashes between the community and military personnel who were there to enforce social distancing as an Ebola response measure [71].

Following the observed rural-urban dynamic, there were county-level distinctions in the Ebola response interventions. Except for Bomi and Margibi counties, where participants reported higher odds for a single response, the other counties reported lower odds for multiple responses. John Stanturf et al. [72] study on social vulnerability and Ebola infection found that counties in the northwestern part of Liberia, including Bomi, Margibi, Lofa, and Montserrado, recorded the highest risk vulnerability to Ebola infection, probably because of population-related dynamics. This vulnerability led to county-level Ebola intervention strategies such as the establishment of community care centers for quarantine services, Ebola education campaigns in and around hospitals, and active Ebola monitoring and prevention initiatives in Bomi and Margibi counties [69,73]. Similar initiatives were undertaken in Lofa and Montserrado counties; however, the Ebola disturbances or riots associated with implementing Ebola control measures might have affected people’s perceived response activities in Montserrado [16]. For people in Montserrado County, military and police surveillance may not have constituted an Ebola response, reflected in participants reporting lower odds for single and multiple responses.

While our study contributes to the literature on the importance of community mobilisation and engagement during disease outbreaks, our analyses and discussion may have contributed to some unintended assumptions, which we note. First, our study is limited to five of the 15 counties in Liberia, so interpretation and generalizability should be limited to these contexts. Second, while we ensured that data collection was of the utmost quality through the quality management strategies we implemented, participant responses may be affected by recall and social desirability bias. Data for this study were collected 8 years after the first Ebola case was detected and 6 years after Ebola ended in Liberia. It is possible that participants may have challenges recalling Ebola response strategies. Third, while it is essential to bring visibility to women’s social reproductive work, especially in a context where patriarchal structures and institutional barriers continue to discriminate against women in social and political forums, we acknowledge that Liberian women face multiple interconnected and structural challenges; as such our analysis and discussion may risk the valorisation and essentialisation of women’s social reproductive and caregiving functions. Traditional gender roles continue to mar women’s gender equality aspirations, with many lagging behind in political representation, formal education, and economic empowerment [74]. Gender-based violence (GBV) is on the rise despite the passage of the 2005 Rape Act and the 2019 Domestic Violence Act [75]. We also acknowledge the work of feminist political economy scholars who have cautioned us about the ‘double burden’ of care work and the feminisation of poverty that comes with valorising women’s care work, particularly in how the ‘caring economy’ benefits capitalist states [29,76,77]. That said, Liberian women’s contribution to health and well-being and the country’s development cannot be refuted. While they are mainly presented as victims of war, there is a changing tide where women are beginning to write their stories by showcasing work that positions them as agents rather than helpless victims [78]. These are the perspectives that inform the presentation of our analysis and discussion.

## Conclusion and policy recommendations

This study underscores the contribution of women’s groups’ presence and mobilising activities to influence Ebola response strategies in Liberia. While local, national, and international partnerships and collaborations significantly contributed to containing Ebola in Liberia, community involvement and grassroots mobilisations have been crucial to this success. The community mobilisation efforts resulting from their trust-based relationships and global visibility benefited the communities in which women’s groups worked. As discussed elsewhere [15], the Government of Liberia and its international partners need to sustain existing community-based partnerships and collaborations while allowing space for new partnerships to develop. Activities of these local partners, including women’s groups, can be streamlined and better resourced to support Liberia’s public health infrastructure. Women’s groups can be trained and deployed to at-risk communities as community health liaisons, which can help with early detection for future pandemic preparedness. The Ebola epidemic in Liberia exacerbated the social and economic vulnerabilities of most people, affecting how they perceived response strategies. The social and economic impacts of Ebola will linger on; therefore, there is a need for a sustained social assistance program to support women and struggling families in their recovery.
